# Cleidocranial Dysplasia: Case Report of Three Siblings

**DOI:** 10.5005/jp-journals-10005-1027

**Published:** 2009-08-26

**Authors:** Rinku Mathur, Manohar Bhat, Satish V, Mohd Parvez

**Affiliations:** 1 Final Year Postgraduate Student, Pedodontics and Preventive Dentistry, Jaipur Dental College, A-87, Manu Path, Shyam Nagar, Jaipur-302019, Rajasthan, India; 2 Professor and Head, Department of Pedodontics and Preventive Dentistry, Jaipur Dental College, Dhand Tehsil Amer, Jaipur- 302101, Rajasthan, India; 3 Reader, Department of Pedodontics and Preventive Dentistry, Jaipur Dental College, Dhand Tehsil Amer, Jaipur-302101 Rajasthan, India; 4 Lecturer, Department of Pedodontics and Preventive Dentistry, Jaipur Dental College, Dhand Tehsil Amer, Jaipur-302101 Rajasthan, India

**Keywords:** Cleidocranial dysplasia, dermatoglyphics, chin cup therapy.

## Abstract

*Background:* A family case report of cleidocranial dysplasia (CCD) with varied manifestations from father to three siblings is presented. CCD ( MIM # 119600) is a rare autosomal dominant skeletal dysplasia caused by CBAF1 gene ( OMIM 600211) with a wide range of variability. In all the cases generalized dysplasia in bone, prolonged retention of primary teeth and delayed eruption of permanent teeth were evident. Interestingly, there were no supernumerary teeth present. There was mandibular prognathism which was intercepted by occipital chin cup therapy.

*Aims and objective:* To present the clinical manifestations, diagnostic imaging and treatment modalities along with dermatoglyphics in CCD patients.

*Conclusion:* Cleidocranial dysplasia is an uncommon disorder however its clinical and radiological features are characteristic. In addition the CCD patients may be distinguished by specific dermatoglyphic markers. It carries with it several implications in terms of complications like skeletal malocclusion, dental caries, etc. Medical treatment is mainly directed at orthopedic and dental correction. A team approach to the management of dental abnormalities on a long-term basis with the overall goal to provide an esthetic facial appearance and functioning occlusion by late adolescence or early adulthood should be focused.

## INTRODUCTION

Cleidocranial dysplasia (CCD) is an autosomal dominant highly polymorphic skeletal disorder with a wide variety of expressivities, primarily affecting bones undergoing intramembranous ossification. It is characterized by retarded cranial ossification, patent sutures and fontanelles, supernumerary teeth, short stature and a variety of other skeletal abnormalities.^1^,^2^

CCD is a rare disorder with a prevalence of less than 1 per million.^3^ The disease gene, which has been mapped to chromosome 6p21 within a region containing core binding factor activity 1 (CBFA1), a member of the Runt family of transcription factors controls differentiation of precursor cells into osteoblasts and is essential for both membranous and endochondral bone formation.^4^,^5^

The different clinical manifestations reflect the basic mechanisms of skeletal development, patterning, bone and cartilage formation, growth and homeostasis.^6^ The oral manifestations of CCD include an underdeveloped maxilla with a high, narrow arched palate, prolonged retention of deciduous teeth, failure of the secondary dentition to erupt, delayed maturation among the permanent teeth and multiple impacted supernumerary teeth.^1^,^7^-^9^

The term dermatoglyphics, is used in describing the scientific fields of study of the palmer and plantar ridges of the hands and feet. Dermal palmer and plantar ridges are highly useful in biological studies. Their notably variable characteristics are not duplicated in other people, even in monozygotic twins or even in the same person, from location to location. The details of these ridges are permanent. Yet while the individual characteristics are variable, that diversity falls within pattern limits that permit systematic classification.^10^

The aims and objectives of this article are to present the dental, radiological and dermatoglyphic findings along with treatment modalities in a family with CCD.

## CASE 1

An 8 years old boy reported to the Department of Pediatric and Preventive Dentistry, Jaipur Dental College with the chief complaint of decayed right lower back tooth. On general examination height and weight was normal to his age. Further examination revealed brachycephaly, frontal bossing and sloping of shoulders. The facial symmetry was normal with oval form, straight profile and competent lips. Intraoral examination revealed the presence of following teeth:

16, 55, 54, 53, 52, 51, 21, 62, 63, 64, 65, 26

46 84, 83, 82, 81, 71, 72, 73, 74, 75, 36

**Fig. 1:**
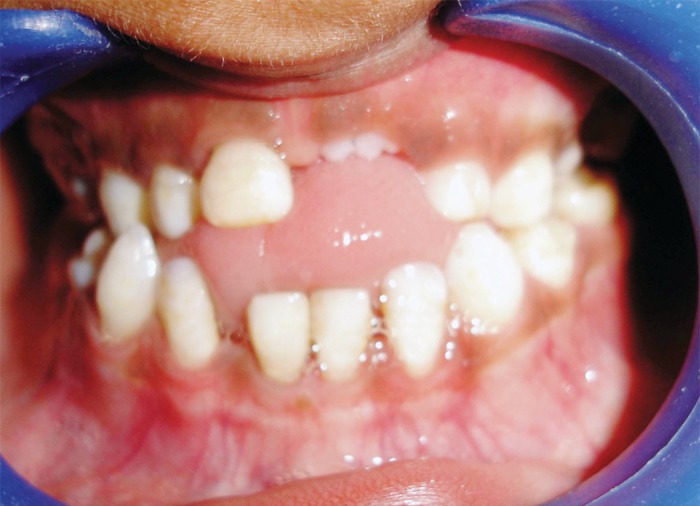
Anterior crossbite

**Fig. 2:**
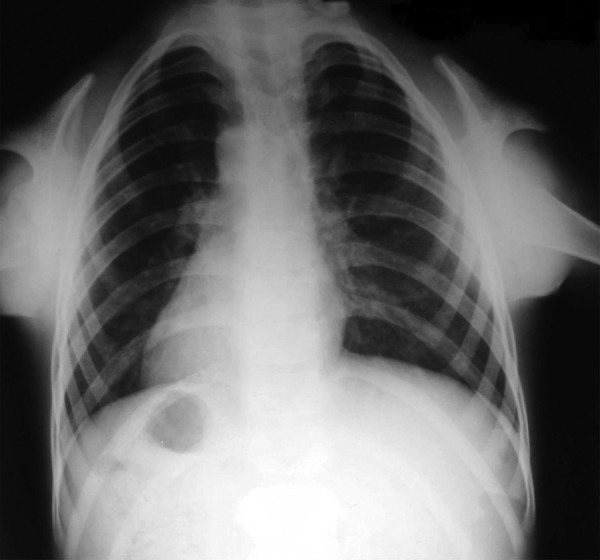
Aplasia of clavicles, bell shaped thorax

**Fig. 3:**
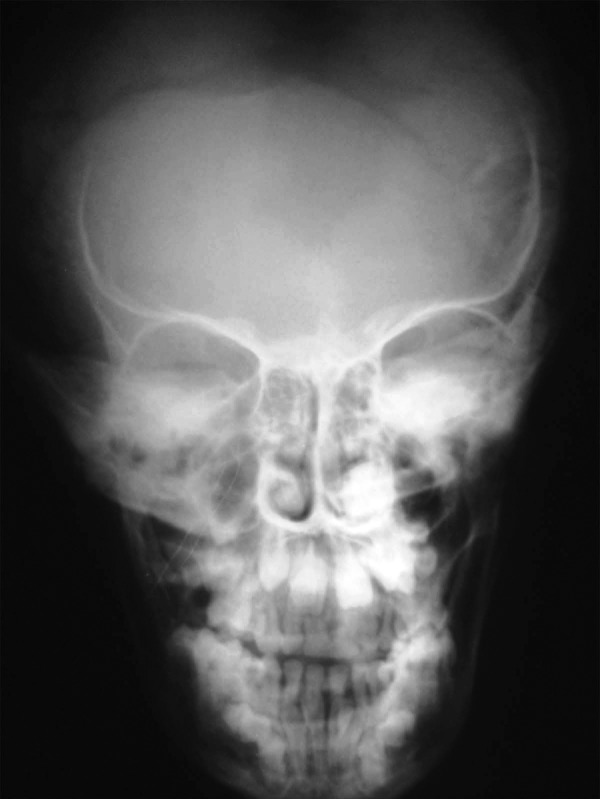
Widening of sutures with few wormian bones

**Fig. 4:**
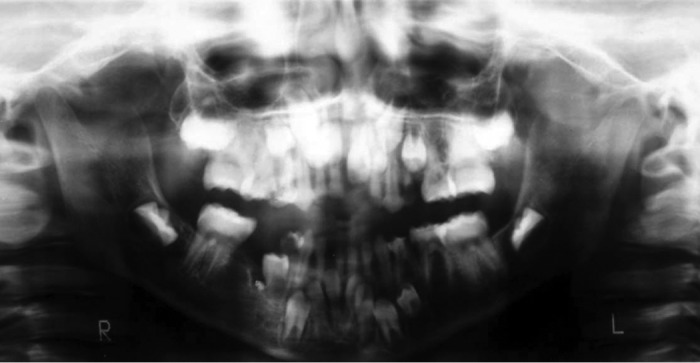
Panoramic view revealing a large number of retained deciduous teeth and missing second premolars

Root Stumps in relation to 64, 84; deep proximal caries in relation to 54, 74 and moderate proximal caries in relation to 55 were observed. The palate was narrow with high vault. On the left side angle’s class III molar relationship was evident whereas on the right side posterior crossbite was observed. In addition the patient exhibited anterior crossbite (Fig. 1). Radiological investigations were planned for the patient. Intraoral periapical radiograph in relation to 54, 74 revealed involvement of pulp. Chest radiograph displayed total absence of clavicles and a bell shaped thorax with low placed scapulas (Fig. 2). A-P view of the skull demonstrated widening of sutures and a few wormian bones (Fig. 3). OPG depicted a large number of retained deciduous teeth coinciding with delayed eruption of the permanent teeth. In addition , the second premolars were missing in both the arches (Fig. 4). The lateral cephalometric analysis confided a wide sella, increased growth axis (Y-axis) as well as increased FMA (Fig. 12). Pulp therapy with stainless steel crowns were luted in relation to 54, 74. Glass ionomer cement restoration was done in 55. The abnormal increased vertical growth pattern of the mandible was intercepted using occipital chin cups (Fig. 13). The dermatoglyphic analysis was also performed. Bilateral arch pattern depicted CWWLL (composite, whorl, loop) sequence type in the left fingers and LCWLL (loop, composite, whorl) pattern in the right fingers. The total ridge count was 71 (Table 1 and Fig. 14). The patient was diagnosed of cleidocranial dysplasia evidenced by clinical and radiographic findings.

## CASE 2

**Fig. 5:**
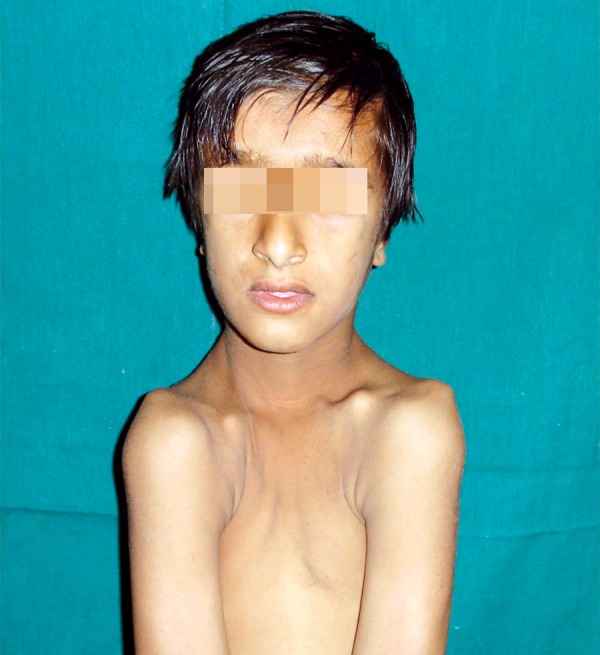
Brachycephaly, frontal bossing, sloping of shoulders and approximation of shoulders towards each other

A 14 years old male, the brother of case1 was investigated in order to detect the genetic predisposition of cleidocranial dysplasia. The patient was short statured, with normal weight and gait. On physical examination the patient had abnormal movement of the right shoulder and the right clavicle demonstrated hypermobility (Fig. 5). Patient showed a straight profile with prominent chin. On intraoral inspection the following teeth were present:

16, 55, 54, 53, 52, 51, 21, 62, 63, 64, 65, 26 46, 85, 84, 83, 82, 81, 31, 72, 73, 74, 75, 36

**Fig. 6:**
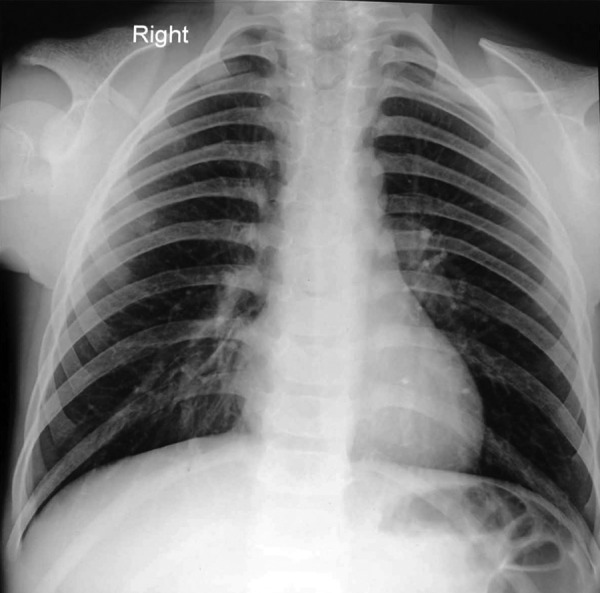
Hypoplastic clavicles and bell shaped thorax

**Fig. 7:**
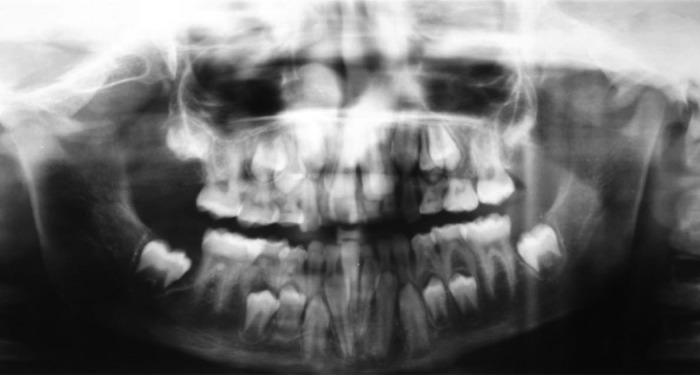
Panoramic view revealing prolonged retention of primary dentition and delayed eruption of the permanent dentition

Occlusal examination pictured angle’s class III malocclusion with bilateral posterior crossbite. High palatal vault with narrow maxillary arch were also apparent. Chest radiograph presented a bell shaped thorax with hypoplastic clavicles (Fig. 6). On A-P view of the skull wide open sutures with open fontanelles and few wormian bones were evident. Lateral cephalometric analysis revealed reduced height of the lower third of the face and a skeletal class III tendency. This could be due to under development of the maxilla and an upward and forward rotation of mandible. This was substantiated by an increased Y-axis and FMA (Fig. 12). On Panoramic radiograph both the arches had over-retained deciduous teeth with unresorbed roots (Fig. 7). The abnormal upward and forward mandibular rotation was intercepted by occipital chin cup therapy (Fig. 13). Dermatoglyphic findings for bilateral arch pattern revealed a specific LLLLL (loop) sequence type on both the hands and a total ridge count of 78 (Table 1 and Fig. 14).

**Table 1:** Table 1: Dermatoglyphic findings in a family with cleidocranial dysplasia

*Name*		*Side*						*Finger*						
				*V*		*IV*		*III*		*II*		*I*		*TRC*
Nandlal (Father)		Lt		L		W		L		W		L		94
		Rt		W		W		L		W		L		
Rahul		Lt		L		L		L		L		L		78
		Rt		L		L		L		L		L		
Mahendra		Lt		C		W		W		L		L		71
		Rt		L		C		W		L		L		
Seema		Lt		L		W		L		L		L		65
		Rt		L		L		L		L		L		
Lt – Left; Rt – Right; L – Loop (Fig. 21 A); C – Composite (Fig. 21 B);														
W – Target whorl concentric circles (Fig. 21 C); TRC – Total ridge count.														

## CASE 3

Another sibling, 11 years old female was also investigated to assess the genetic relationship of cleidocranial dysplasia. She was of average height and weight with a normal gait. examination revealed retained primary teeth and delayed eruption of permanent successors which is one of the characteristic feature. The teeth present were:

16, 55, 54, 53, 52, 51, 61, 62, 63, 64, 65, 26

46, 85, 84, 83, 82, 81, 71, 72, 73, 74, 75, 36

Anterior and a unilateral right posterior crossbites were present. The radiographic view of the chest revealed aplasia of clavicles and low placed scapulas with a funnel shaped thorax (Fig. 9). The A-P view of skull concluded wide open sutures and fontanelles along with multiple wormian bones (Fig. 10). The hypoplastic appearance of maxilla was clearly evidenced on the lateral cephalogram. The malrelationship between maxilla and mandible could arise through discrepancies in the effective horizontal lengths of both the arches or due to an abnormal contribution by the projected length of the cranial base element. The projected lengths were measured and mandibular length which exceeded the standard indicated mandibular prognathism. Vertical parameters pointed the increased Y-axis along with an elevated FMA (Fig. 12). These findings ascertained a relative mandibular prognathism. The oral pentamogram findings were partial anodontia with the absence of 14, 15, and 25 (Fig. 11). This relative mandibular prognathism was intercepted by occipital chin cup therapy (Fig. 13).The dermatoglyphic findings were LWLLL (loop, whorl) and LLLLL (loop) sequence patterns in left and right hands respectively. The total ridge count was 65 (Table 1 and Fig. 14).

**Fig. 8:**
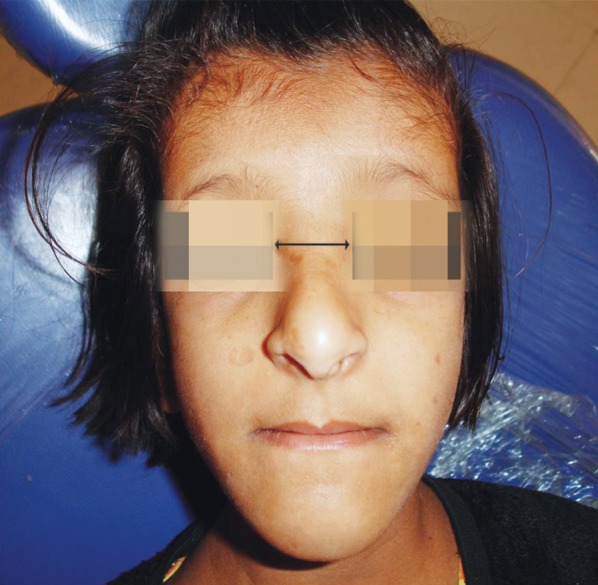
Depressed nasal bridge, frontal bossing, brachycephaly and hypertelorism

**Fig. 9:**
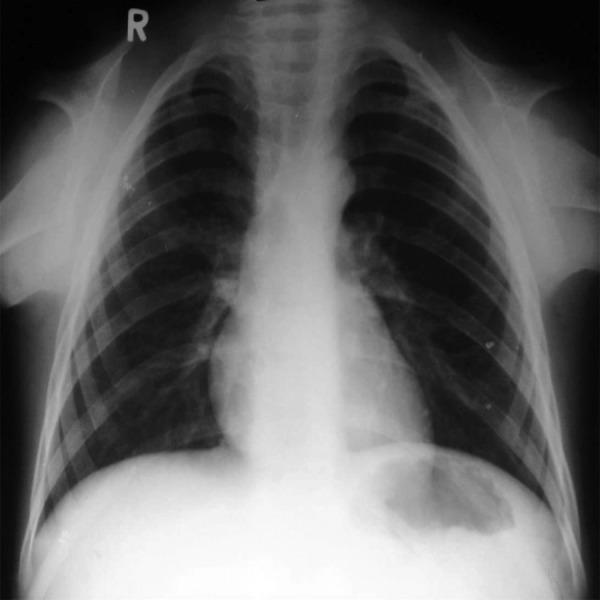
Aplasia of clavicles and funnel shaped chest

**Fig. 10:**
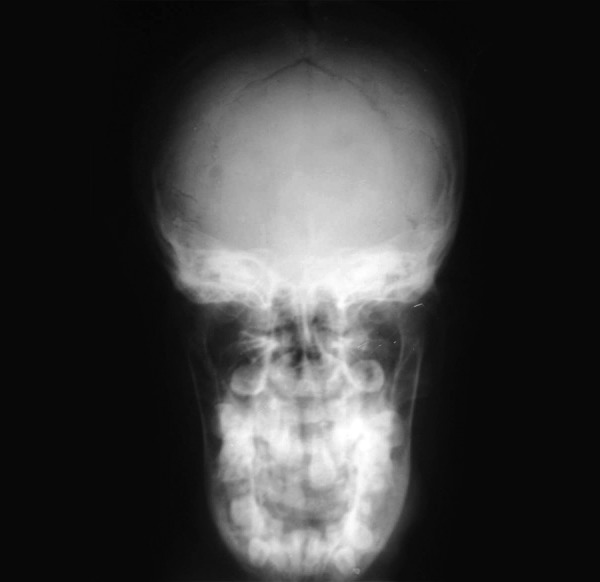
Wide open sutures and fontanelles, multiple wormian bones

**Fig. 11:**
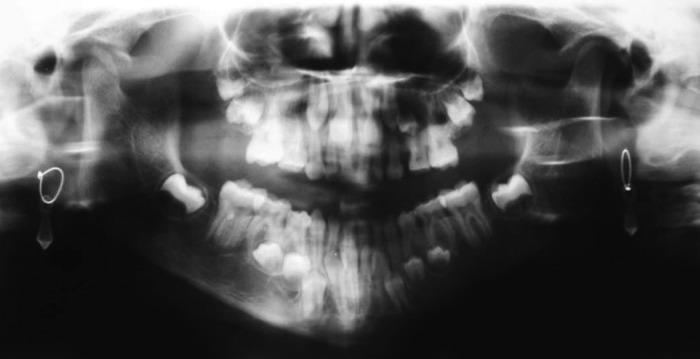
Panoramic views revealing partial anodontia

**Figs 12A and B:**
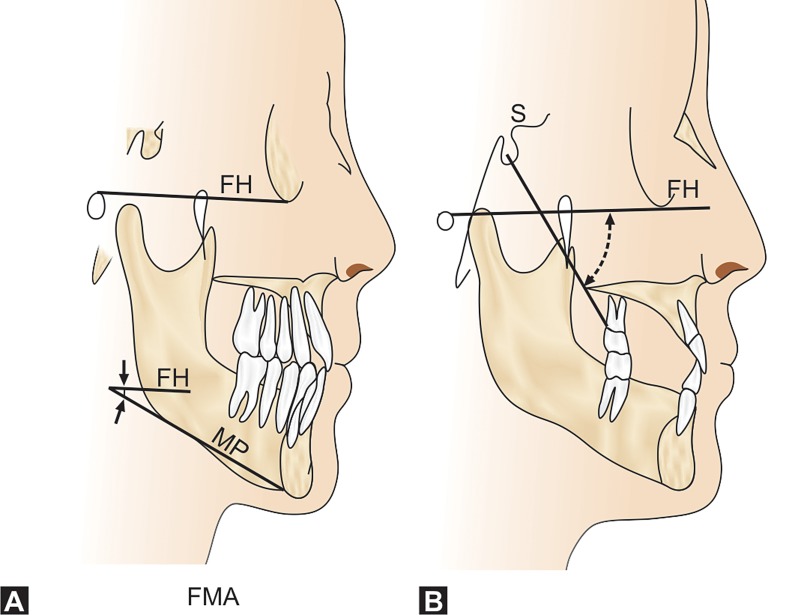
(A) FMA – frankfort mandibular plane angle; FH – frankfort horizontal plane; MP – mandibular plane (B) Y-axis (Growth axis) FH – frankfort horizontal plane; S – sella, Gn – gnathion

## DISCUSSION

CCD is an autosomal dominant disorder of bone caused by a defect in CBFA 1 gene and represents a generalized dysplasia of skeletal structure. Being genetic in nature the disease may pass generation to generation as any other asset. Cleidocranial dysplasia is characterized by abnormalities of the skull, teeth, jaws and shoulder girdle as well as by stunting of the long bones. The defect of the shoulder girdle from which the condition drives its name ranges from complete absence of clavicles in about 10% to partial absence or even simple thining of one or both clavicles. Patients with cleidocranial dysplasia characteristically exhibit a high, narrow arched palate. The maxilla is usually underdeveloped and smaller in relation to mandible. One of the outstanding oral findings is prolonged retention of deciduous teeth and susequent delay in the eruption of succedaneous teeth.^11^

**Figs 13:**
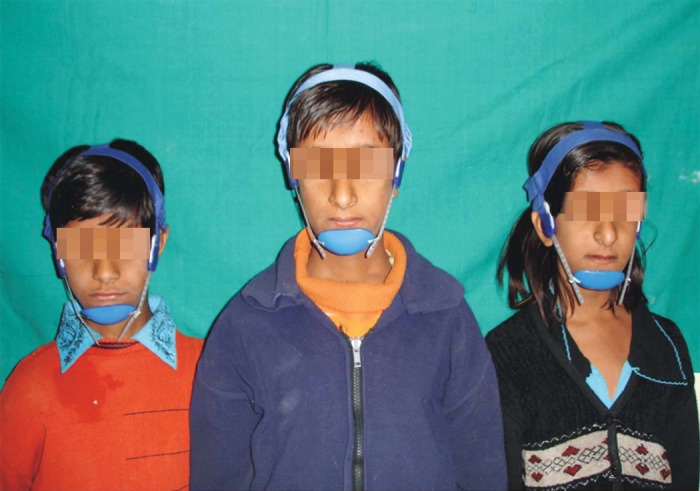
Occipital chin cup therapy in case 1,2 and 3

A rare family case of CCD reported in our department that was not previously diagnosed, though the patients had a history of prolonged retention of primary teeth and a history of extractions as a result of dental caries .The relatives of the cases resided in another state due to which there was paucity of investigations for those relatives. The mutational analysis of RUNX2 was not carried out as proposed by Yoshida et al.^12^ It is possible that the Run domain was affected since the patients showed short stature. Supernumerary teeth are considered to be a diagnostic feature of CCD. However complete absence of supernumerary teeth and hypodontia is reported in all the three cases presented. Yoshida^12^ pointed out that there is a significant correlation between the supernumerary teeth and short stature with the gene dosage RUNX2 effect in the RUNX2 activity. In the present clinical cases, the number and the severity of alterations were different in each patient. RUNX2 controls the maturation of both osteoblasts and odontoblast. Therefore a delay in tooth maturation is expected in RUNX2 deficient tissues. This is reflected in the clinical situations, where the dental maturation of CCD subjects is retarded by as much as 4 years.^7^,^13^,^14^

The findings of CCD, although present at birth, could be easily missed due to its low frequency and variety of clinical manifestations which were evident in our cases too. The clinical and radiological studies revealed slow growth and moderately short stature. The dysplasias may include various combinations of absence, lack of fusion, or incomplete modeling of any of the 3 ossification centers in each clavicle or in the right one alone. This is evidenced by the aplasia of clavicles in cases 1 and 3 along with hypoplasia of the right clavicle in case 2. Other clinical findings included low placed scapulas and deformities of the thorax. The intraoral inspection showed unresorbed roots and prolonged retention of primary teeth. In addition to this, there was absence of some permanent teeth, high palatal vault and anterior cross bite. The primary teeth erupted on time however; the subsequent permanent teeth exhibited a delayed eruption, presumably as a result of defective eruption pathway. The first permanent molars erupted spontaneously in all the patients which could be ascribed to firstly, a very thin layer of bone to pass through for these molars and secondly, their eruption is not dependant on root resorption of deciduous teeth.^15^ In the canine and premolar regions the persistence of primary teeth and delayed root resorption hindered the eruption of successors. The teeth were shaped regularly with no structural anomalies.

The radiologic features of this disorder are very characteristic. With respect to the skull it is the membranous portion and not the base that is affected. As such, delayed ossification leads to delayed closure of sutures (sagittal and coronal) and fontanelles (metopic).^16^ The cephalometric features of the cases displayed increased Y axis and marked increase in the FMA. These irregular changes resulted in mandibular rotation (Y axis-FH), ramus inclination –FH and the mandibular plane (FH) being forwarded with a clockwise rotation, causing a mandiblular protrusion.^17^ ^18^ The large number of unerupted teeth in the premaxilla and mandibular symphysis regions makes identification of points A and B, commonly used to represent the anterior limits of the dental bases, difficult. The results by the cephalometric analysis confirmed the clinical reports of mandibular prognathism in all the cases. This could be attributed to a large anteroposterior mandibular length together with a shortened cranial base.^18^ The interventional occipital chin cup therapy was given to all the cases as they were indicative of increased Y axis and FMA. The cases are under regular follow-up. This interception would prevent the further worsening of the Class III malocclusion.

**Figs 14A to C:**
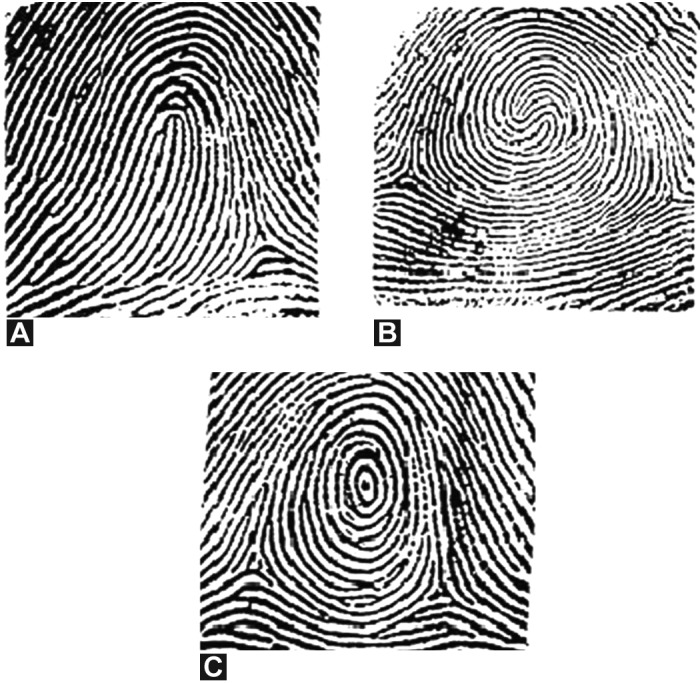
Dermatoglyphic findings. (A) Loop, (B) Composite, (C) Target whorl concentric circles

Dermatoglyphic ridge patterns have been widely studied in major malformation syndromes. In the present case report, total ridge count (TRC) and bilateral arch patterns were examined in the father and 3 siblings. The dermatoglyphic findings of these CCD patients with respect to the TRC was found to be lower than the normal study population in the region.^19^ The cases exhibited varied arch patterns and different sequence types. There was a predominance of Loop pattern in all the cases (Fig. 14A). Interestingly, the eldest son, case 1, demonstrated Loop pattern in all the fingers. The other arch patterns were Composite (Fig. 14B) and Target whorl concentric circles (Fig. 14C). Specifically no composite pattern was found in the case 3. This hints that the formation of ridges and TRC as a marker are influenced by genetic differences. It also indicates some genetic association between CCD patients and fingerprint patterns. CCD may be genetically associated with loop, whorl and composite patterns which can be further investigated by detailed molecular studies. Thus, although the present study is based on a very small number of individuals, it does indicate that the CCD patients may also be distinguished on the basis of dermatoglyphic markers.

*What this paper adds?*

1. Early diagnosis and intervention in CCD patients may prevent a number of problems and create good esthetic and functional results.
2. The dental problems if intervened before adulthood can prevent skeletal malocclusions like short lower facial height and mandibular prognathism.
3. Dermatoglyphic findings may be an auxiliary tool in distinguishing the CCD patients.

*Why this paper is important for pediatric dentist?*

1. The treatment plan is largely dependant on both the chronological and dental age of the patients.
2. The timing of diagnosis in CCD is not only important in choosing an appropriate treatment plan but also in attaining a successful result.
3. Multidisciplinary approach should be planned by dental, pediatrics, orthopedics and genetic counseling team.
